# Collaborative Processes in Science and Literature: an In-Depth Look at the Cases of CERN and SIC

**DOI:** 10.3389/frma.2020.592819

**Published:** 2021-01-13

**Authors:** Emilia Leogrande, Renato Nicassio

**Affiliations:** ^1^ European Organization for Nuclear Research (CERN), Geneva, Switzerland; ^2^ Independent Researcher, Bari, Italy

**Keywords:** collaboration, collaborative process, research collaboration, collaborative novels, scientific collaboration analysis

## Abstract

In this paper we examine how the process of collaboration works in science and literature. In the first part, we discuss the features of scientific collaboration and literary collaboration and the differences between them. In the second part, we analyze two processes of collaboration, each from a different field: the case of CERN and high-energy physics and the case of Scrittura Industriale Collettiva and its Great Open Novel. Lastly, we try to compare those two processes and deduce the common traits of a successful collaboration.

## Introduction

The word “collaboration” comes from the late Latin verb *collaborare* which meant to work jointly with someone else. Thus, etymologically speaking, collaboration is the act of working together. However, working together could mean very different things depending on the field in which the action takes place. To collaborate in a string ensemble is not the same as to collaborate in a soccer team. The starting point might be the same–more people doing something together–but then everything changes: the number of persons involved, the organization of their tasks, the scope they have to fulfill. As a matter of fact, collaboration is much more than a plural number of people working together. It is a complex process whose features and functions vary dramatically not only from field to field but also from case to case. Like Tolstoy’s unhappy families, each collaboration–be it happy or unhappy–is a collaboration in its own way. But this does not mean that collaborations cannot be compared or that collaboration as a subject *per se* does not exist. In fact, seen as a process, each collaboration cannot help showing some constants. In order to analyze them, in this paper we have approached the subject of collaboration from two distinct perspectives: science and literature. The choice is no accident. The scientific and the literary fields are probably the different fields *par excellence*, the ones where one could hardly expect to find common traits. Indeed, as to collaboration, they seem to be on the opposite sides of the barricades.

In science collaboration seems to be the rule and it is regarded as a positive feature as well as an inevitable one. In literature collaboration seems to be the exception and, when established, arises doubts that seem to question its own legitimacy. Thus, in the first part of our paper we investigate motives and reasons of this apparent contrast. In the second part we analyze two processes of collaboration, each from a different field: the case of CERN and high-energy physics and the case of Scrittura Industriale Collettiva and its Great Open Novel. Lastly, we compare those two processes in order to underscore the differences and try to isolate the common traits of a successful collaboration.

## Science: Collaboration as the Rule

According to Y. N. Harari, the first achievement toward scientific progress was a collective admission of ignorance. By agreeing on the fact that humans do not hold the answers to fundamental questions, humankind as a whole felt the urge to fill the gap:

“Modern-day science is a unique tradition of knowledge inasmuch as it openly admits collective ignorance regarding the most important questions.” (Harari, 2011: 279–281).

The common need to find explanations and rules set the stage for the development of a universal batch of methods and tools. The scientific method, defined as the process by which science is carried out, is founded on the concepts of universality and reproducibility which are valid throughout space and time. Like all processes, the scientific process undergoes improvements and refinements over time. However, the key to its success is its unique capability of adjusting and tweaking itself in order to retain its effectiveness. From this perspective, it may be well argued that the scientific process is the main product of modern science, the main invention of modernity:

“Our greatest invention in the past 200 years was not a particular gadget or tool but the invention of the scientific process itself. Once we invented the scientific method, we could immediately create thousands of other amazing things we could have never discovered any other way. This methodical process of constant change and improvement was a million times better than inventing any particular product, because the process generated a million new products over the centuries since we invented it. Get the ongoing process right and it will keep generating ongoing benefits. In our new era, processes trump products.” (Kelly, 2016: 6).

In charge of this ongoing process there is not a single person nor a specific group of persons. It is the scientific community as a whole that oversees and fosters the scientific process. Thus, one could say that the scientific community plays the role of one big collaboration in which all researchers work ideally toward the common goal of advancing knowledge. Collective ignorance, as Harari would put it, asks for collective work.

However, this broad definition of science as collaboration lacks to grasp the detailed complexity and variety of nowadays scientific process. As of today, the scientific process is collaborative at many and interrelated levels. Not only do researchers work together as members of a global community where all findings are pooled and made available to anyone. They also work together in a more concrete way: as fellows of a same laboratory, participants of a same experiment, partakers of a same project. Moreover, researchers may be part of different teams at the same time. The result is a multiplicity of groups of very different size–from below ten to thousands–that somehow all refer to other groups. If in our new era processes trump products, within the processes, collaboration trumps individualism. In contemporary science, no matter the discipline, to research means to collaborate. Those who work in the field do not separate the concepts and usually talk of “research collaboration.”

Research collaboration is a recent trait of science. In 1963, in his famous essay *Little Science, Big Science*, Derek de Solla Price argues that the trend toward increasing collaboration represents «one of the most violent transitions that can be measured in recent trends of scientific manpower and literature» (De Solla Price, 1986: 79). Later studies confirm the trend and indicate the increase of collaboration as one of the critical features that best distinguishes premodern from modern science. Collaboration in science grows at a remarkable rate from 1980 to 2000, skyrocketing in the 1990s (Doré et al., 1996; Georghiou, 1998; Adams et al., 2005), and continues to rise as of today both in terms of people and nations involved (Bornmann et al., 2015; Wagner et al., 2015). The tendency can be seen across all fields of science (Wagner et al., 2017) but of course the quality and quantity of research collaboration do differ by discipline. Although there is no consensus on what discipline should be regarded as the more collaborative, it is generally agreed that the rate of collaboration is higher in experimental disciplines such as Physics and Biology where the sharing of data and instrumentation constitutes the base of research, and lower in pure theoretical ones such as Mathematics where the simultaneity of data access and the use of large and expensive equipment are not always required (Newman, 2001; Wuchty et al., 2007; Mattsson et al., 2008). There are multiple and often connected reasons that account for the large increase of scientific collaboration in the last sixty years. The list of potential contributing factors is endless but Katz and Martin (1997) provide a useful summary:

“1) Changing patterns or levels of funding; 2) The desire of researchers to increase their scientific popularity, visibility and recognition; 3) Escalating demands for the rationalization of scientific manpower; 4) The requirements of ever more complex and often large-scale instrumentation; 5)Increasing specialization in science; 6) The advancement of scientific disciplines which means that a researcher requires more and more knowledge in order to make significant advances, a demand which often can only be met by pooling one's knowledge with others; 7) The growing professionalization of science, a factor which was probably more important in earlier years than now; 8) The need to gain experience or to train apprentice researchers in the most effective way possible; 9) The increasing desire to obtain cross-fertilization across disciplines; 10) The need to work in close physical proximity with others in order to benefit from their skills and tacit knowledge.” (Katz-Martin, 1997: 4).

Factors 1-3-4-5–6. are of particular interest since they underline a fundamental issue: collaboration in science has become a need much more than a choice. The escalating costs of facilities and instrumentation, the subsequent tendency of funding agencies to encourage partnerships, the growing complexity and variety of abilities required, all point out the necessity of collaboration. Factor 7., although considered by Katz and Martin less important at present times, is depicted by Beaver and Rosen (1978) as a sort of comprehensive explanation for scientific collaboration. «Scientific collaboration represents a response to the professionalization of science [...] Professionalization refers to a dynamical organizational process which led to a revolutionary restructuring of what had been a loose group of amateur and full-time scientists into a scientific community” (Beaver and Rosen, 1978:65–66). Thus, according to them, professionalization holds for both the historical development and the current growth of collaborative research, in contrast to specialization, which represents a more recent phenomenon and a partial response to professionalization, as it does not justify the variation in the incidence of collaboration by field, even at the same level of specialization. Another factor that has to be taken into account in the last years is the spread of the Internet and information technologies. Thanks to them collaboration is way more simpler and wider and this contributes to reinforce its importance and presence in science. And its presence is today so strong and common that C. Wagner and L. Leydesdorff can see research collaboration as an international «self-organizing system creating a network of relationships that can be observed at the communication levels» (Wagner and Leydesdorff, 2005:8): a sort of meta-field with its norms and culture that permeates all fields of sciences and that is responsible for its own existence and growth. This might sound hyperbolic but, when you think about it, the success of research collaboration was part of science since its very beginning. The universality of the laws of Nature together with the reproducibility of the scientific process implicitly mustered the potential for collaboration and all that followed was precisely that potential put to good use.

## Literature: Collaboration as the Exception

The literary field is quite a different place. Collaboration is far from being the rule both in research activity and, even more so, in creative practice. Skimming through the products of literature–from academic journals to novels–collaboration seems to be a rare and somewhat odd event, an exception to a rather individual habit.

As a discipline and area of studies, literature has a strong tradition of individualism. Though often linked to schools and movements, critics and scholars usually act as soloists. They refer to the works of others, talk and write about others, but seldom work with others. Besides, when they do so, they tend to work within much smaller teams compared with what generally happens in science: two or three people against tens or even thousands. On a first level, this difference can be easily explained by practical reasons. Unlike most of scientific research, research in literature does not require large and expensive tools. Collaboration is therefore a choice rather than a need. But on a deeper level there are issues of methodology and ideology that make collaboration–and especially long-term and large-scale collaboration–a quite complicated enterprise in the literary studies. Whereas the scientific community builds itself around the scientific method, the literary studies have no universal method to refer to. As a consequence, literary critics and scholars may follow different approaches thus lacking a common ground in which collaboration might be rooted. Moreover, in those approaches, subjectivity has still a big role to play. A literary research is always, at least in part, a matter of interpretation whose value depends on the eyes of the interpreter.

All this makes research collaboration not only difficult and therefore uncommon but also sometimes discouraged. In a field built by–and around–individuals, a regular process of collaboration would require a profound transformation of long-established patterns of practice and assessment: resistance must be taken for granted. Lisa Ede and Andrea Lunsford, two literary scholars who have been working together for years, point out that «despite vigorous debates over theories and methods surrounding issues of subjectivity and authorship, ideologies of the individual and the author have remained largely undisputed in scholarly practice» (Lunsford and Ede, 2001: 358). As of today, the single-author book is still «a virtual necessity for promotion and tenure in most research universities» (*ibidem*) and there are humanities conferences that do not even allow co-authors to present (Siemens, 2015). This prominence of the individual in the literary studies is strictly related to the prominence of the individual in their subject. To study literature means to study the works of individuals, that is works conceived and composed by singular and specific subjects. Thus, in a way, the solitary researcher of literature can be seen–or perhaps sees himself–as a reflection of the solitary author of literature. In his preface to *The Rules of Art*, Pierre Bourdieu investigates the reasons as to why literary scholars exert such a «resistance to scientific analysis» and his conclusions blame precisely the tempting parallelism that is often carried out between the singularity of the work of literature, the singularity of its creator, and the singularity of its critic, all seen as *individui ineffabili*, singular entities that cannot be rationally explained nor compared to something else, and therefore–one could add–cannot collaborate with someone else. In Bourdieu’s scornful words:

“Why such implacable hostility to those who try to advance the understanding of the work of art and of aesthetic experience, if not because the very ambition to produce a scientific analysis of that *individuum ineffabile* and of the *individuum ineffabile* who produce it, constitutes a mortal threat to the pretension […] of thinking of oneself as an ineffable individual, capable of ineffable experiences of that ineffable? Why, in short, such resistance to analysis, if not because it inflicts upon “creators,” and upon those who seek to identify with them by a “creative” reading, the last and perhaps the worst of those wounds inflicted, according to Freud, upon narcissism, after those going under the names of Copernicus, Darwin and Freud himself?” (Bourdieu, 2010: 5).

Bourdieu’s emphasis on the egotism of “literary people” might sound like a simplistic explanation. However, it would be hard to deny that literature, understood as a creative practice, is probably the egotistical art *par excellence*. By now the romantic figure of the solitary genius has been exposed as a myth (Stillinger, 1991) and literature itself has been variably interpreted as a social construct in which the creative power of the individual is tied to the collective culture he or she happens to live in. But nonetheless novels and poems do remain the domain of subjectivity. The author of the literary text is–or at least appears to be–a single person, and a relation of reciprocal uniqueness is commonly established between them with the structure and meaning of the text seen as dependent on the style and intention of the author. *Mutatis mutandis*, the same holds true for literary criticism: what a paper says, and how it says it, cannot be easily distinguished nor separated from the person who says it. The effect is a sort of reversal of what usually takes place in science. The main focus is not on the process but on the product, i.e. the text, seen as the specific result of a specific mind. The process, on the other hand, is often hidden and, in Bourdieu’s words, somehow ineffable, which of course does not constitute a fertile ground for collaboration. After all, the literary process is basically writing–to work, in literature, is to write–and writing is perhaps one of the most private and individual acts humans do. A collaborative process of writing would require a sharing of tasks that are deeply intertwined and on which it could be hard to reach a consensus, especially when it comes to literature. The planning of a story, the creation of narrators and characters, the choice of words, the building of rhythm, the relation between form and content… Those are just some of the events that take place before and during an act of creative writing. Most writers would deem them unsuitable for collaboration and as a matter of fact most writers do not collaborate. To write a poem or a novel together is definitely not a need and it might look like a challenge more than a choice. However, challenges as well as exceptions do exist.

## Describing a Scientific Collaboration: The Case of CERN and High-Energy Physics

### What is a Scientific Collaboration?

Research collaboration in science is usually measured by bibliometric analysis. The number of multiple-authored (or co-authored) publications is used as a counting unit to measure collaborative activity: the higher the number, the wider the collaboration. Strictly speaking, however, this method does not measure the act of collaboration but its output. A paper that lists the names of twenty authors is a sure sign of collaboration but it says little about the nature of its process. It does not tell how it started, why it started, what course it followed, what task distribution took place, etc. Even the scale itself might have been different. Some of the authors might have been cited for social and academic reasons but did not actually participate in the research (Hagstrom, 1965). Or maybe there were people who contributed informally–perhaps making an enlightening comment during lunch–and whose contribution was not recognized. Thus, the collaborative activity could be both overestimated and underestimated. Melin and Persson (1996) used co-authorships to study research collaboration but they acknowledged that co-authorship data are «a rough indicator of collaboration» and that «a certain level of uncertainty» is inevitable (3). As Katz and Martin (1997) conveniently summarize, «bibliometric analysis of multiple-author papers can only be used as a partial indicator of collaborative activity» (3). As Katz and Martin (1997) point out, «bibliometric analysis of multiple-author papers can only be used as a partial indicator of collaborative activity» (3). Collaboration is not a plural number of people who publish together. It is a field of relations that are not always easy to see and track. Almost any attempt to define it would omit something and end up being unsatisfying. For instance, one could define scientific collaboration as a group of people providing inputs to a particular piece of research. However, this would raise the question of what an input is. Should it be quantified in terms of time spent on the research? And if so, are we supposed to measure time in relation to its quantity (hours spent working on a project) or its quality (results achieved)? And what about money? Isn’t financial support a contribution, and a fundamental one, to research activity? Moreover, a collaboration may involve subjects that are not people in a strict sense. Katz and Martin (1997) recognize different levels of collaboration depending on the nature of the subjects involved–individual, group, department, institution, sector, nation–and distinguish an intra form of collaboration (when it occurs within a same level) from an inter form (when it occurs between different levels). To complicate things further, the profile of a collaboration may change over time making its complex dynamics even harder to follow. On account of this, we decided to focus our attention on collaboration in “mega-science projects” in which the size of the endeavor asks for a definite assignment of tasks and roles. A mega-science project involves hundreds of experts of different fields and requires large-sized equipment and big amount of investment. Although not representative of the entire pool of scientific disciplines, mega-science projects share interesting collaborative features and by now represent a common trend in many of the sciences. As the knowledge demands push the technological requirements to the limit, international projects for big science become more and more needed and widespread. The case of CERN is paradigmatic.

### A Mega-Science Project

**FIGURE 1:**
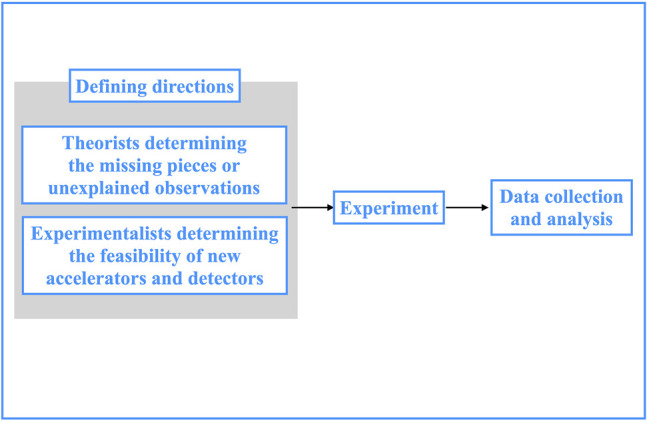
General workflow of the process of scientific collaboration at CERN.

CERN, an acronym for Center Européen pour la Recherche Nucléaire, is an intergovernmental organization founded with the prime aim of performing fundamental research in nuclear physics. Situated in Geneva, Switzerland, it hosts the world’s most powerful particle accelerator, the Large Hadron Collider (LHC), which makes protons and ions collide in four points of its 27 km circular tunnel. As of 2017, more than 17.500 people from around the world work together on this project. CERN’s staff members, i.e. people employed directly by CERN, number around 2.500. The remaining members are affiliated with institutes in more than 70 countries, for a total of 110 nationalities. Such big community is organized in two interrelated categories: Experiments and Collaborations. An Experiment is a project aimed at pursuing a dedicated scientific goal and it includes the hardware and software components required to fulfill it. A Collaboration is a group of scientists–ranging in size from below ten to thousands–who propose, design, build and operate an Experiment and have free access to its data. The biggest Experiments at the four colliding points of the accelerator (named ALICE, ATLAS, CMS and LHCb) come with Collaborations that number 1.500, 3.000, 4.000 and 800 people circa respectively, including scientists, engineers, technicians and administrative personnel. Each Collaboration has an internal structure of working groups that carry out various and diverse tasks. One’s membership to a Collaboration is usually dictated by defined rules that leave little space to personal interpretation. For example, PhD students who carry out their work within an Experiment have to perform a service-task duty in addition to their doctoral work for a definite amount of time (typically 6 months) in order to gain authorship rights, i.e. to sign all the publications issued by the Collaboration. In line with Beaver and Rosen (1978), collaboration «becomes a mechanism for both gaining and sustaining access to recognition in the professional community». The ultimate scope of these Collaborations is help answering fundamental questions of this kind: what is the Universe made of (matter), and what keeps it together (forces)? The investigation begins with what might be termed a creative step: deciding which kind of experimental steps to pursue and defining directions to do so. All this is accomplished by collecting in theoretical models all available information–in the form of theoretical hypotheses or experimental results–and pinpointing the missing pieces or the unexplained observations. This task is typically steered by theorists who work side by side with experimentalists. Nowadays debate is focused on the post-LHC facilities: which particles will we have to collide to find what LHC has not been able to reveal? And at which energies? LHC has found the Higgs boson, but no sign yet of any other particle that would solve unexplained mysteries of the Universe. Experimentalists perform studies on the feasibility of new accelerators and detectors, assessing their performance through simulations. In this phase, prototypes are built and tested with particle beams available at the present machines, to optimize and validate their designs. A huge amount of R&D is dedicated to achieve technological, methodological and computational performance beyond the current capabilities, and this is how the creativity developed in fundamental research often translates into scientific advance in everyday life. Once the required performance is proved to be achievable, the construction in full scale is realized. Thus, when the accelerator is ready to collide particles and the experiments are ready to detect the products of the collisions, twenty years of scientific development have already been pursued. The data taking process (online monitoring of the collisions, storage of data collected, …) is followed by the analysis of the data and subsequent interpretation of the results. The latter phase is pursued again in close cooperation with theorists. The general workflow of the process of scientific collaboration at CERN is illustrated in Figure 1. For such a complex organization to work, it is fundamental to guarantee an efficient system of information exchange. In fact, each Collaboration has its own communication system that is established through the so-called Collaboration Weeks–in which ongoing work for the different research topics is presented to all members–and lower-level working group meetings. In this way, the information flows top-down and bottom-up within the hierarchical structure of the Collaboration. Of course, communication tools are largely exploited to this end. The world wide web (WWW) itself, born at CERN in 1989, was originally conceived and developed to meet the demand for automatic information-sharing between scientists in universities and institutes around the world. Today, the average physicist relies heavily on e-mail services and on the use of chat platforms as well as videoconferencing tools. As pointed out by Shrage (1995), the benefits of technological tools on how people think and collaborate are countless (one can follow ongoing discussions, keep track of good ideas and objections, solve problems and conflicts) and these are indeed largely exploited at CERN. However, in spite of its strong collaborative and communicative environment, CERN scientific research profits also from some degree of competition among Collaborations, typically from the ones that share the same scientific goals. ATLAS and CMS, for instance, were both involved in the search for the Higgs boson, whose discovery was announced conjunctively in 2012. Yet, during the investigation, the data collected by the two Experiments were restricted to the members of the corresponding Collaboration: results were made public only when validated within it. Thus, a non-trivial system of protecting and sharing information is at play among different Collaborations. Conferences are usually the places where private results are shared as preliminary but a plethora of technical workshops, topical conferences and dedicated gatherings across Collaborations accompany the scientific process, often with less-stringent privacy requirements. As a consequence, developments in technologies and algorithms, adapted at one’s own case, are not uncommonly inspired by other people’s work.

The ultimate output of this collaborative process is, of course, the publication of the results. As said above, publications are just a partial indicator of collaborative activity and still their importance cannot be overlooked: the scientific publication is the place where both the investigation and findings of the scientific process are scrutinized and communicated. Moreover, writing a scientific paper is a collaborative effort as well. As a matter of fact, a scientific paper at CERN is typically written by the ones who have directly contributed to the subject of the publication and the subject of publication can be related to any phase of the process: the theory that lead to the experiment in the first place, the technical development (hardware and software) and the analysis of the results. On average, the theoretical papers are written by a handful of people whereas the technical reports are usually produced by larger groups since they require a more diverse expertize. As for the analysis papers, the number of authors can vary much depending on the complexity of the particular topic, but in many cases they represent the outcome of a PhD work, thus they are often written by the students and supervisors. After the drafts are completed by the authors, they undergo a complex iterative process of reviews, edits, comments and corrections by a large community of experts. Normally, the first review involves the conveners of the working group whose job is to validate the scientific quality of the publication. Once their comments are implemented, the draft is sent to a Publication Committee. The size of this organ is proportional to the dimension of the Collaboration. For small Collaborations it can include four or five people. For larger ones such as ATLAS and CMS it can even include twenty people. The expertize of the board is heterogeneous in order to respond to the different subjects addressed in publications. The Publication Committee is also in charge to organize the editorial process within the Collaboration: the publication is circulated among all members of the Collaboration for a period of circa two weeks during which comments and reviews can be sent by the members requiring editorial changes or further scientific validations. At the end, when the paper is re-submitted to the Committee, all comments must be successfully addressed, either as changes in the paper itself or in an official way. To this end, CERN provides a centralized repository in which documents can be uploaded, comments and reviews can be added and whole discussions can take place without losing traces. At this stage, papers are mature enough to be submitted to arXiv, a repository of electronic preprints (known as e-prints) approved for posting after moderation but not full peer review. Depending on the target journal, the peer review can take weeks to months, in which the paper can undergo further edits and improvements. Thus, when finally published, the paper has been through of numerous revisions that guarantee that the high standards of scientific research are successfully met. No official record of the people who wrote it in the first place is kept. In the process, the structure of the Collaboration is clearly defined but, in the product, any individual contribution is made–on purpose–impossible to trace from outside.

## Describing a Literary Collaboration: The Case of SIC and the Great Open Novel

### What is a Literary Collaboration?

As with science, describing a literary collaboration is no trivial task either. In “The Art and Mystery of Collaboration” (1890), one of the first studies about the subject, Brander Matthews openly admits the difficulty: telling how a literary collaboration works is «at best but a doubtful possibility» (Matthews, 1901: 315). Indeed, as the title of his essay suggests, it is almost a mystery. In broad strokes, a literary collaboration might be defined as a process involving multiple people for the production of a literary text. But saying so only skims the surface of a much more complex affair. To begin with, in producing a literary text–be it a novel, a poem or a play–everything from form to content is opened to debate and subject to constant change. Thus, a literary collaboration will hardly be a tidy sequence of neatly divided phases. Instead, it will be a composite, simultaneous and recursive process. To complicate things further, discussing a literary collaboration from the outside is not always a possibility. In most cases, all we see of a literary collaboration is its product: the text. The only people who really know the process behind it are the participants themselves but their trustworthiness or even their willingness to talk cannot be taken for granted. Sometimes writers who collaborate refuse to talk about their own process of collaboration because they see it as a devaluation of their work. It is the case of Edith Somerville who dismissed the persistent curiosity regarding her collaboration with Martin Ross questioning: «How does anyone write if it comes to that?» (London, 1999: 108) In her opinion, their collaborative writing was equal to writing tout court and any question about it was therefore pointless if not disrespectful: their novels, like all novels, should interest for what they said and not for how they were created. When asked about her life-long collaboration with Michael Dorris, Louise Erdrich’s answer was kinder but equally elusive: «It’s a sort of a conversational process; we just talk about it all the time» (Karrel, 2002: 33). They just talked. Their collaboration just happened.

This lack of precision is instrumental. The less the collaborative process of literature is explained, the less it appears as a process that can be explained. Thus, it may become closer to what it is usually intended as literary creation: a quite ineffable act whose importance does not lie in its functioning but in what gives birth to. This happens especially when the scale of collaboration is quite small, as is with Somerville and Ross or Dorris and Erdrich, only two people working together. At least in theory, two writers may hide their collaborative process quite easily: as a couple, they may disguise it behind usual and indescribable factors such as the affinity of taste, the existence of a sentimental bond, the presence of a constant dialogue. In his aforementioned essay, Matthews points out that the first requisite to a literary collaboration that involves two partners is a «sympathy» between them and that collaboration itself might be likened to «matrimony» (Matthews, 1901: 315–316).

However, when the scale of collaboration gets larger, things change. A process of literary collaboration that involves four, six or more writers cannot pretend it does not exist. Four, six, or more writers cannot be likened to a married couple nor write as they talk. They must follow a procedure that can be explained. In fact, in many collaborative texts written by large groups of people, there are pages that fully describe the process that has been followed. For instance, in Six of One by Half a Dozen of the Other (1872), an American novel by six writers, there are six prefaces that tell almost everything about how the text was produced, from plot invention to chapter distribution. The same happens, more than a century later, in Keeping Mum (2014), an English novel by fifteen writers, that provides readers with an afterword that explains in detail how the collaborative novel came to be written. The list of examples could keep going regardless of time and space. Explanatory paratexts can be found also in Las vírgenes locas (1886) a Spanish novel written by eleven writers, Le Roman de Quatre (1923), a French novel written by four, Lo zar non è morto (1929) an Italian novel written by ten, Caverns (1990) an American novel written by fourteen. Of course, compared with what we have seen happened at CERN, those groups of writers are still quite small. It is a matter of perspective. In a field dominated by individuals, a team of ten people looks like a crowd. Yet, even in literature, there are collaborations that can be objectively labeled as large-scale. It is the case of In territorio nemico, an Italian novel that is the product of a collaboration that counted more than a hundred people. In a way it might be regarded as a literary equivalent of a mega-science project. In the following, we discuss its process.

### A mega-literary project

In 2007 Italian writers Gregorio Magini and Vanni Santoni presented SIC, an acronym for Scrittura Industriale Collettiva, “Industrial Collective Writing.” As the name itself partly suggests, SIC was a collaborative process aimed at organizing the creativity and writing of a group of people through a peculiar division of tasks and labour that, in a way, might resemble a factory. But SIC was much more than a factory of writers. Magini and Santoni described it as both a «method and a community» (Magini and Santoni, 2008) and as a matter of fact SIC was also an online community where all people interested in the method could discuss it and participate in its experiments. The idea of SIC has its roots in Magini and Santoni’s desire to change the supposed uniqueness of literature. In the last decades, in almost any field of human knowledge, the way people build cultural contents depends more and more on technology and collaboration. Magini and Santoni saw no good reason why literature should keep being an exception. Thus, they devised SIC to employ the advantages of technology and collaboration in order to produce literary texts. SIC main ambition was to produce a Great Open Novel, «a collective book written by hundreds of people» (Magini and Santoni, 2008). That book is the aforementioned In territorio nemico, a historical novel set in the Italian Resistance during World War II, which came out in 2013.

The decision to write a historical novel was up to Magini and Santoni and it was no accident. They thought it was particularly apt for a community that had to work together: «We were […] struck by a glaring analogy: if writing a historical novel necessarily involves working with a system of external sources, one could say, taking the suggestion to the extreme, that every historical novel is by definition a form of collective writing» (Magini and Santoni, 2011). If the choice of the genre depended on Magini and Santoni, the building of the plot was performed in an open and collaborative way. A sort of online call for papers was launched asking everyone to send stories and anecdotes of events occurred during World War II in Italy. The only requirement was that these materials had not yet be coded by historiography: they had to be something personal or belonging to the memory of a family. At the end of this first step, forty-one war stories were collected from which Magini and Santoni outlined a general plot for the novel and presented it to the SIC community that was now ready to start. The SIC community was based on a hierarchical yet dialogical mode of organization. There were roles with different levels of duties and responsibilities but, on the website, there was a free and open debate through which, at least potentially, anyone could bring changes to the entire process. The SIC roles were essentially two: Writers and Composers. Writers wrote the different parts that would create the text, each writer working aside from the others. Composers supervised Writers’ work and collected their individually-written parts in order to mesh them together and create a collective text. Those parts, however, were not consecutive temporal sequences. In territorio nemico was not made up of different chapters written by different writers and then combined by a team of editor-bricoleur. The writing of the novel was carried on by dozens of people following thematic principles and, in the end, no part of the novel was conceived and written by a single person.

According to SIC, the creation and the crafting of a story can be parceled out in its structural elements–such as characters, locations, actions, and so on–before the story itself exists. Those elements can be extrapolated from a general plot and addressed in a scheda, a specific file named after its specific subject. As for In territorio nemico, characters and locations were the first files that had to be completed. There were twenty-four character files corresponding to twenty-four different characters of a novel that had yet to be written: The Matteo File, the Adele file, the Aldo file... Every file had between four and eight available slots, depending on its importance. The Writers who were interested in a particular file could reserve it, write down their own version and then pass it to the Composers who were in charge of that specific file processing. Thus, at this point there could be, for instance, eight different versions of the character of Matteo–a writer may have described him as slim and tall, another as fat and short; he might have been shy and generous for someone and arrogant and selfish for someone else, and so on. The Composers had to read all those versions, operate a selection of their most interesting and suitable parts, and combine them in a new collective file that could be traced back to no one of its actual creators.

Of course, given their faculty to dissect others’ texts and generate new ones, the Composers did exert a remarkable control on the overall process. Their number was way smaller than the Writers’–there were eight Composers and seventy-eight Writers–but their power was greater. At the same time, however, the Composers had to work with Writers’ words and ideas only. They were not allowed to add new images. Moreover, the overall process tended to favour community over individuality: if a certain idea recurred in two or more individual files, the Composers were bound to accept it in their final file.

**FIGURE 2:**
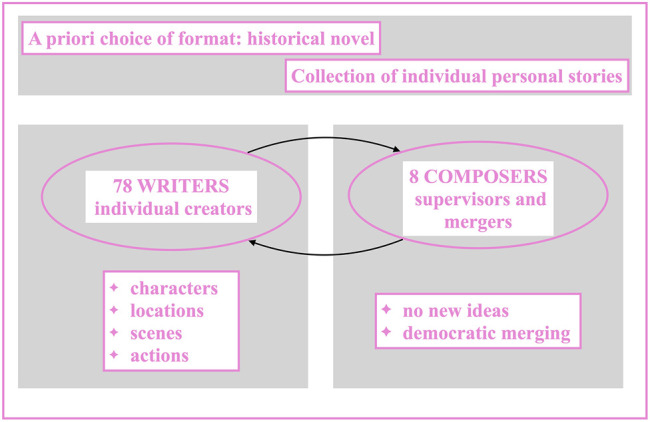
Illustration of the collaborative process at SIC.

This hybrid process of individual work and collaborative outcomes took place for all the characters and the locations of the novel in progress. Once completed, the Composers forwarded the new files back to the Writers who now had to work with materials that had no longer a personal nature but a collective one. Thus, the drafting began. Writers had to follow a general elaboration of the story named treatment which was built in the same way as characters and locations and which provided a description of every scene of the novel. Once again, more writers worked separately on a same scene, each group on a different scene. They developed its structure, built its form. Then they passed their personal versions–yet now based on multi-personal materials–to the Composers who re-composed them in new files, sent them back to the Writers, and so on till the novel was completed. The whole process took around fifteen months. It produced 935 individual files corresponding to around 3,000 pages from which 170 final collective files were obtained–24 character files, 35 location files, 18 treatment files, 93 drafts (an illustration of the collaborative process at SIC can be found in Figure 2). The resulting book has 308 pages and each page underwent so many cycles of planning, writing, composing, editing, that it is impossible to find a single page that is the product of a single mind and hand. The book is the product of a collaborative process in which each individual contribution disappears among others.

### Comparison: The Antelope and the Campfires

Two processes of collaboration, two very different aims: finding answers to the unresolved mysteries of the Universe and inventing a story that can be told in a novel. What could they possibly have in common? Very little, as Carlo Rovelli seems to suggest in the final chapter of his bestselling essay Seven Brief Lessons on Physics:

“When we talk about the Big Bang or the fabric of space, what we are doing is not a continuation of the free and fantastic stories which humans have told nightly around campfires for hundreds of thousands of years. It is the continuation of something else: of the gaze of those same men in the first light of day looking at tracks left by antelope in the dust of the savannah–scrutinizing and deducting from the details of reality in order to pursue something which we can’t directly see but can follow the traces of.” (Rovelli, 2015: 74).

Science is connected to the human need to understand and master the surrounding environment. Like the prehistorical hunt for the antelope, scientific research means pursuing and explaining something that exists and whose traces can be found and followed. Literature, on the other hand, is connected to the human need to invent and tell stories–and, of course, listen to them. It means creating and narrating something that does not exist and whose features can be determined during its own creation. The difference is great and can be observed in the analyzed cases since their very beginning. At first, the imaginative process at the bottom of literary collaboration is almost boundless. Before writing, the members of the SIC community were free to propose ideas for the plot and imagine their own version of characters, locations, events, etc. without any significant limit. However, as writing began, they were forced to follow schemes and consider each other’s thoughts and choices. Their personal imagination was tempered with a collective one: they had to compromise. Literary collaboration, then, seems to put restraints on the individual freedom to imagine and write in order to produce a common text. Moreover, especially when established in large-scale fashion, it cannot often grant full authorial recognition. All the people who contributed to the SIC novel had their names listed at the end of the book. An authorship of this kind is extremely unusual and therefore not commonly recognized. If you wrote a book along with dozens of people, you are not regarded as a real author. Professionalization in literature does not rhyme well with collaboration. All this might help explain the rareness of literary collaboration. Who writes doesn’t want restraints, at least not for a long time, and aspires to gain a name for himself or herself. Apart from *In territorio nemico*, the SIC community never produced another collaborative novel and, in general, most groups of people who collaborate in literature do not last: they work together to compose a text but once it is accomplished they usually go back to their individual occupation.

In science, it is quite the opposite. The imaginative process at the bottom of scientific collaboration has many boundaries but it is rather enduring. When they formulate hypotheses and imagine solutions, physicists at CERN are tied to respect fundamental theoretical laws. Moreover, the feasibility of conducting an experiment to prove or disprove those hypotheses has to be taken into account. A good idea, then, is not enough to start a collaborative process, at least in experimental science. It has to be practically workable. It may take years to decide what insight may be worth pursuing and even more years to build the required tools. In this sense, collaboration is extremely useful to overcome obstacles both of “software” and “hardware” nature. The more people try to fix a problem or fill a void, the higher the probabilities of success. Moreover, all the participants in a scientific collaboration can expect to gain a professional advantage from it: their role, be it in the workplace or in a paper, is recognized inside and outside the actual group and therefore it can be used job-wise. Thus, whereas literary collaboration seems to exert a restraining power on the individuals involved, scientific collaboration may end up increase their capabilities. It is then no accident that, unlike literature, it happens on a daily basis. Every day since 1954, hundreds of people gather at CERN to do research within its international model of collaboration.

Given all those differences, scientific and literary collaborations should be two incomparable activities and in fact, under many aspects, they are. At the same time, however, if we focus on the collaborative process itself and not on its scope, object, and method, scientific and literary collaborations do show some interesting comparable features. To begin with, as collaborative processes, they both involve groups of people and groups cannot help having–and displaying–some kind of structure:

“There is no such thing as a 'structureless' group. Any group of people of whatever nature coming together for any length of time, for any purpose, will inevitably structure itself in some fashion. The structure may be flexible, it may vary over time, it may evenly or unevenly distribute tasks, power and resources over the members of the group. But it will be formed.” (Freeman, 1972).

Freeman is right. When more people come together for some time, a structure is inevitable. But when more people work together for some time, a structure is not only inevitable but also indispensable. Any collaboration that has a goal to fulfill–be it a novel to write or a particle to detect–needs a structure, that is, a hierarchy. This is shown both in CERN Collaborations and in the SIC community. In both cases a task distribution takes place assigning different levels of responsibility and power. Each person has a job to perform and must refer to a supervisor and a set of rules. All this prevents, or at least reduces, the dangers of competition and chaos that are embedded in any collaboration. A group of people who work together sharing ideas, efforts, and sometimes the same workplace, constitute a rather unstable element. Rivalry might arise between participants turning collaboration into antagonism. Moreover, unexpected problems of communication and organization might jeopardize the collaborative endeavor introducing disorder in the system. Thus, a group can always degenerate into a crowd. The presence of a hierarchical structure serves as a precautionary antidote. Although sometimes it may be constrictive, it keeps collaborators–scientists or writers–on the right track and allows them to focus on their objective. Sometimes this structure may be constrictive, thus, in order to be really effective, it has to retain some kind of flexibility. Both CERN and SIC collaboration retain a capability of adjusting themselves as necessary and they both guarantee a certain degree of debate among participants.

As said above, the objectives of the SIC community and CERN Collaborations are deeply different making the beginning and the development of their collaborative processes almost impossible to compare. Yet, at the end of the collaborative process, they both present a written text. Of course, a historical novel and a scientific paper do belong to different categories but once again, if we focus on their structural elements rather than on their contents, those texts reveal some common traits. Both SIC novel and CERN papers, for example, undergo several cycles of writing, revision, editing, and re-writing. Today those are mandatory steps for every text that aspires to be published but, when a text is the product of a collaborative process, those steps logically protract, tangle, and multiply. Theoretically, all the persons involved in the collaboration might have objections or suggestions that must be taken into account. The crafting of the text, then, has to synthetize all the heads behind it and, judging from its final form, also their voices. Both SIC novel and CERN papers conceal the plurality of the process that has produced them. The text is the result of a collaborative effort but in its pages we don’t see nor hear all the people who sustained it. It is a matter of communicability. In the process, the presence of a large number of people can be an advantage in terms of inputs, perspective, and time-saving. In the product, however, it can be a problem. A text written by tens of people each one keeping his personal voice might end up being illegible. Some kind of synthesis seems to be required and both the SIC novel and CERN papers opt for it: the number of their authors is plural but the form of their writing is not. Those texts are written in what might be termed a neutral, rather objective, style. Of course, this could also be a flaw especially for a literary text. A novel, unlike a scientific paper, is not a pragmatic text and its aim goes beyond communication. This is probably another reason why collaboration is not as frequent in literature as is in science: a collaborative literary text is not only hard to produce but it might be a product without a great appeal. In his essay, Matthews argues that collaboration is apt for those literary genres that are based on a «deliberate scientific construction»: genres, that is, where « clearness is needed, where precision, skill, and logic are looked for, where we expect simplicity of motive, sharpness of outline, ingenuity of construction, and cleverness of effect» (Matthews, 1901: 305). Those are features that literary texts do not always present or want to present. The features of the process and of the product for CERN and SIC are summarized in Table 1 and Table 2.

**TABLE 1:** Features of the collaborative product at CERN and SIC.

	Beginning of the process	Method	Effects on individuals involved	Frequency
CERN	Many boundaries of theoretical and practical nature	Universal scientific method	Empowering of singular capabilities;	Standard MO, with almost no exceptions
			Professional advantages, authorial recognition	
SIC	Few boundaries of theoretical and practical nature	Case-dependent method	Constraints and compromises;	Isolated case
			Few/none professional advantages, few/none authorial recognition	

**TABLE 2:** Features of the collaborative product at CERN and SIC.

	Group-structure	Flow of communication	Produced text
CERN	Clear hierarchy Task distribution	Constant	Several cycles of writing, revision, editing, and re-writing
		Flexible	Synthesis of styles and contributions
		Structured	Clear and objective
SIC	Clear hierarchy Task distribution	Constant	Several cycles of writing, revision, editing, and re-writing
		Flexible	Synthesis of styles and contributions
		Structured	Clear and objective

## Conclusion

When we started to work at this paper we had few ideas and many prejudices about collaboration, all rooted in our respective fields. At first, the task was hard. However, as time went by, we began to realize that the subject we wanted to investigate was somewhat close to what we had to do. In order to analyze and compare such diverse processes of collaboration we had to analyze and compare our own process of collaboration. That was the turning point. A process of collaboration, no matter the field or the number of people it involves, needs a structure to organize its plurality. This structure implies a path to follow, a task division, a constant coordination and control of participants’ contributions. The work is parceled, distributed, and continuously judged and adjusted. A product of a collaborative process, no matter the nature or content, is always the outcome of its structure. Yet, in order to be comprehensible from outside, it has to recompose it in a more unified and organic form. SIC and CERN collaborations do it. We tried too.

## Author Contributions

All authors listed have made a substantial, direct, and intellectual contribution to the work and approved it for publication.

## Conflict of Interest

The authors declare that the research was conducted in the absence of any commercial or financial relationships that could be construed as a potential conflict of interest.
